# Supplementary material for: Cellular and molecular characterization of multiplex autism in human induced pluripotent stem cell-derived neurons
Source: Mol Autism. 2019 Dec 30;10:51. doi: 10.1186/s13229-019-0306-0 (PMC6936127; doi:10.1186/s13229-019-0306-0)
Supplement: Supplementary file 2 — Additional file 2: Figures S1-S8. Figure legends provided in file. [file 13229_2019_306_MOESM2_ESM.pdf]

Additional file 2. Legends and Supplemental Figures S1-S8.

Legends for Supplemental Figures

Supplemental Figure S1. Characterization of iPSCs. (A) Exome sequencing identified a heterozygous missense variant in the FAD domain-encoding region of the *GPD2* gene (chr2: 157352686 (hg19) G>A, NM_001083112.2 c.233G>A, p.G78E) in individuals in the pedigree under study, including the unaffected mother (UM), intermediate phenotype sister (IS), and affected proband (AP), as well as in the trait-affected brother (TB) not studied here. **(B)** This variant was confirmed by Sanger sequencing and was not present in an unrelated, unaffected control (UC). **(C-E)** Expression of markers of pluripotency was assessed by (C) RT-qPCR and (D) immunocytochemistry (representative images shown), comparing the cell lines under study (n=4 biological replicates from one clonal line for each subject). GPD2 protein level was also assessed in these iPSC lines by (D) immunocytochemistry or (E) Western blotting (n=2 biological replicates from one clonal line for each subject). **(F)** Both the UC line and clonal lines 1 and 2 derived from the UM, IS, and AP were karyotypically normal. **(G-H)** iPSCs were stained with propidium iodide and analyzed by FACS for DNA content analysis. (G) Representative FACS plots are shown. (H) Mean percentages of cells in each cell cycle stage, with no differences between cell lines observed (n=4 biological replicates from one clonal line for each subject). p-values: *$P<0.05$ were determined by one-way ANOVA in (H) or by unpaired t-test for RT-qPCR in (C), and all other pairwise comparisons had a non-significant p-value ($P\geq0.05$). In (D), scale bar=200μm.

Supplemental Figure S2. Characterization of iPSC-derived cExN NPCs. (A-C) After 12 days of differentiation, cExN NPCs were stained with propidium iodide and analyzed by FACS for DNA content. **(A)** Schematic of cExN NPC differentiation protocol. **(B)** Representative FACS plots. **(C)** Mean percentages of cells in each cell cycle stage (n=4 biological replicates from one clonal line for each subject). **(D-E)** cExN NPCs were plated in equal numbers for each sample

and were counted after four days of culture (n=6 biological replicates from one clonal line for each subject). Data are quantified in (D), and representative images are shown in (E) (scale bar = 300µm). **(F)** GPD2 protein levels were detected in cExN NPC samples by Western blotting (n=2 biological replicates from one clonal line for each subject). p-values: **P*<0.05 were determined by one-way ANOVA and all other pairwise comparisons had a non-significant p-value (*P*≥0.05).

Supplemental Figure S3. Maturation of cExN NPCs. (A) Differentiation scheme, including timeline and small molecules used. **(B)** Immunocytochemistry for proteins marking cExN specification, differentiation, maturation, and neuronal function (scale bar=50µm, n=1 biological replicate from one clonal line for each subject; representative images are shown).

Supplemental Figure S4. Characterization of iPSC lines during differentiation into cIN NPCs (A) iPSCs were differentiated for 12 days, as shown in the schematic. (**B-C**) Differences in neurosphere size at day 5 and 12 are shown in (B) and quantified at day 5 in (C) (mean±SEM; scale bar=250µm; n=7 total biological replicates from two clonal lines for each subject, and one clonal line for the UC). p-values: ****P*<0.001 were determined by one-way ANOVA and all other pairwise comparisons had a non-significant p-value (*P*≥0.05).

Supplemental Figure S5. Transcriptome analysis of cExN and cIN NPCs by RNA-seq. (A) Multidimensional scaling plot for the cExN and cIN NPC samples. **(B-C)** Numbers of differentially expressed genes obtained for each pairwise sample comparison, indicating up- and down-regulated genes for the (B) cExN NPCs and (C) cIN NPCs (p<0.05, fold-change >2).

Supplemental Figure S6. **Networks of DEGs with expression that is similar in the AP/IS and different than the UM.** Networks of DEGs are related to **(A)** cExN **'**neurological disease' **(B)** cIN **'**nervous system development and function', and **(C)** cIN **'**neurological'. Within each

network, red symbols indicate upregulated genes and green symbols indicate downregulated genes, where the color intensity represents relative degree of differential expression.

Supplemental Figure S7. Co-expressed DEGs in cExN and cIN NPCs were defined by hierarchical clustering and visualized on a heatmap. Log2-CPMs of DEGs were scaled by z-score, as described in the methods, and were plotted on a heatmap for **(A)** cExN NPCs and **(B)** cIN NPCs.

Supplemental Figure S8. XIST mRNA expression levels and variancePartition analysis. **(A-B)** Comparison of XIST expression levels in cINPCs derived from the UC, UM, IS, and AP by (A) RNA-seq and (B) RT-qPCR analysis. For the UM, IS, and AP, the first clonal line from each subject was used for RNA-seq analysis (n=4 biological replicates), and the second clonal line from each subject was used for RT-qPCR (n=3 biological replicates), confirming findings for both clonal lines. The single UC clonal line was evaluated by both RNA-seq and RT-qPCR, which also yielded similar results. **(C)** VariancePartition analysis was conducted on RNA-seq data as described in the Methods, with violin plots indicating the percent of variance that was attributable to the cell type, the individual subject from which the model was derived, their age, or their sex.

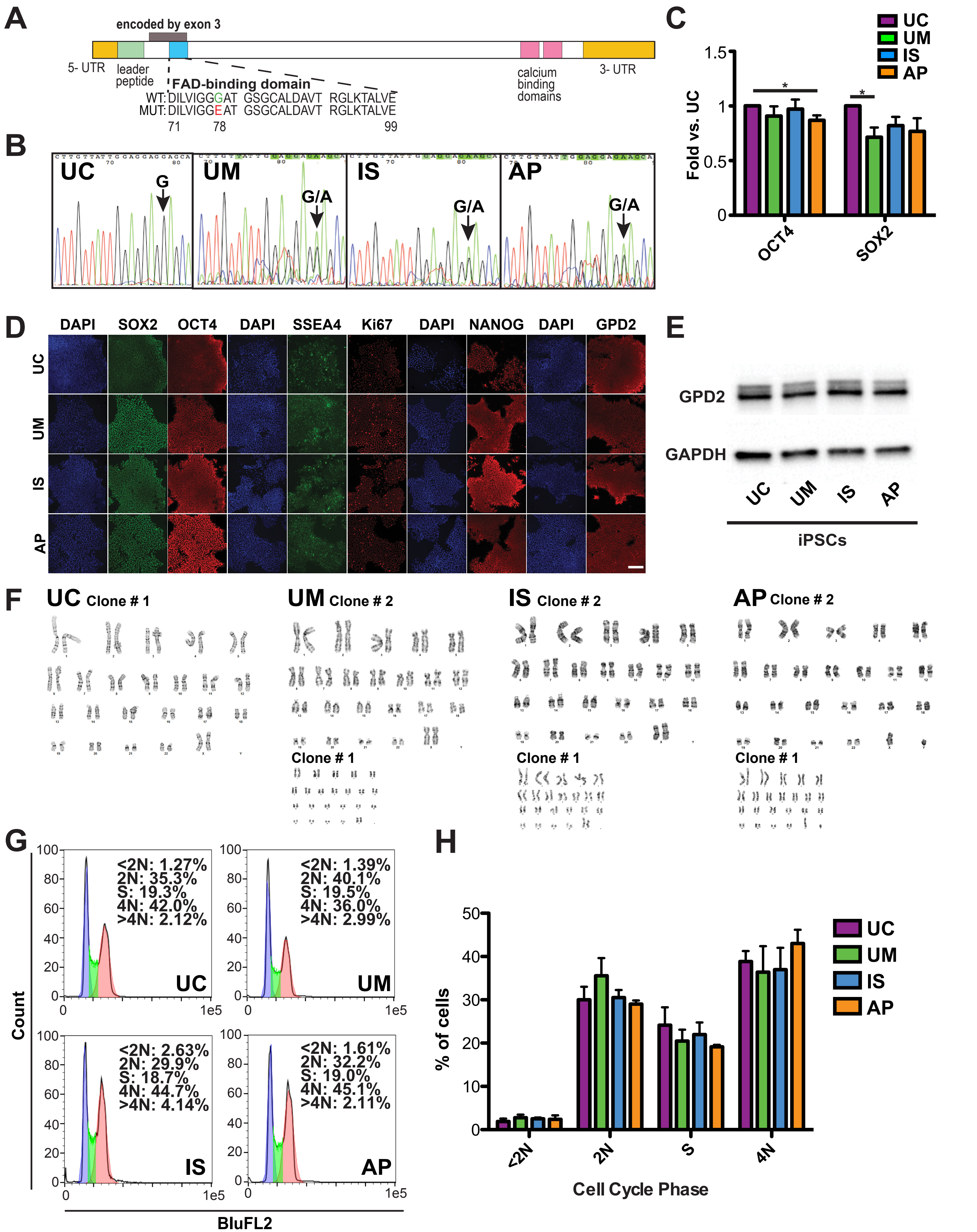
A
encoded by exon 3
5- UTR
leader peptide
FAD-binding domain
WT: DILVIGGGAT GSGCALDAVT RGLKTALVE
MUT: DILVIGGEAT GSGCALDAVT RGLKTALVE
71 78 99
calcium binding domains
3- UTR
B
UC
G
UM
G/A
IS
G/A
AP
G/A
C
UC
UM
IS
AP
Fold vs. UC
OCT4
SOX2
D
DAPI
SOX2
OCT4
DAPI
SSEA4
Ki67
DAPI
NANOG
DAPI
GPD2
UC
UM
IS
AP
E
GPD2
GAPDH
UC
UM
IS
AP
iPSCs
F
UC Clone # 1
UM Clone # 2
Clone # 1
IS Clone # 2
Clone # 1
AP Clone # 2
Clone # 1
G
<2N: 1.27%
2N: 35.3%
S: 19.3%
4N: 42.0%
>4N: 2.12%
UC
<2N: 1.39%
2N: 40.1%
S: 19.5%
4N: 36.0%
>4N: 2.99%
UM
<2N: 2.63%
2N: 29.9%
S: 18.7%
4N: 44.7%
>4N: 4.14%
IS
<2N: 1.61%
2N: 32.2%
S: 19.0%
4N: 45.1%
>4N: 2.11%
AP
Count
BluFL2
H
% of cells
<2N
2N
S
4N
Cell Cycle Phase
UC
UM
IS
AP

S. Fig. S2

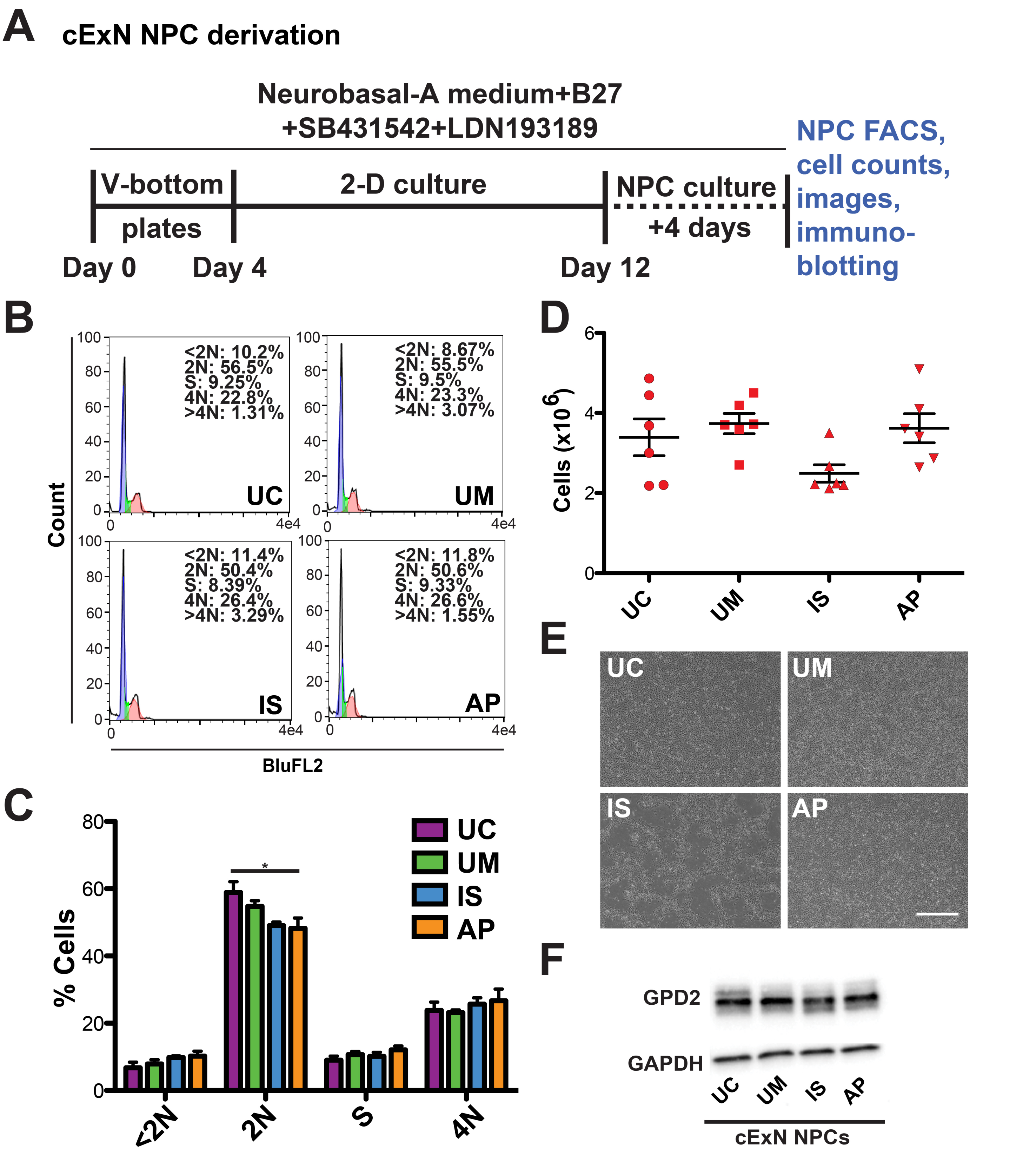
A
cExN NPC derivation
Neurobasal-A medium+B27
+SB431542+LDN193189
V-bottom
plates
2-D culture
NPC culture
+4 days
Day 0
Day 4
Day 12
NPC FACS, cell counts, images, immuno-blotting
B
Count
<2N: 10.2%
2N: 56.5%
S: 9.25%
4N: 22.8%
>4N: 1.31%
UC
<2N: 8.67%
2N: 55.5%
S: 9.5%
4N: 23.3%
>4N: 3.07%
UM
<2N: 11.4%
2N: 50.4%
S: 8.39%
4N: 26.4%
>4N: 3.29%
IS
<2N: 11.8%
2N: 50.6%
S: 9.33%
4N: 26.6%
>4N: 1.55%
AP
BluFL2
C
% Cells
UC
UM
IS
AP
<2N
2N
S
4N
D
Cells (x10^6)
UC
UM
IS
AP
E
UC
UM
IS
AP
F
GPD2
GAPDH
UC
UM
IS
AP
cExN NPCs

S. Fig. S3

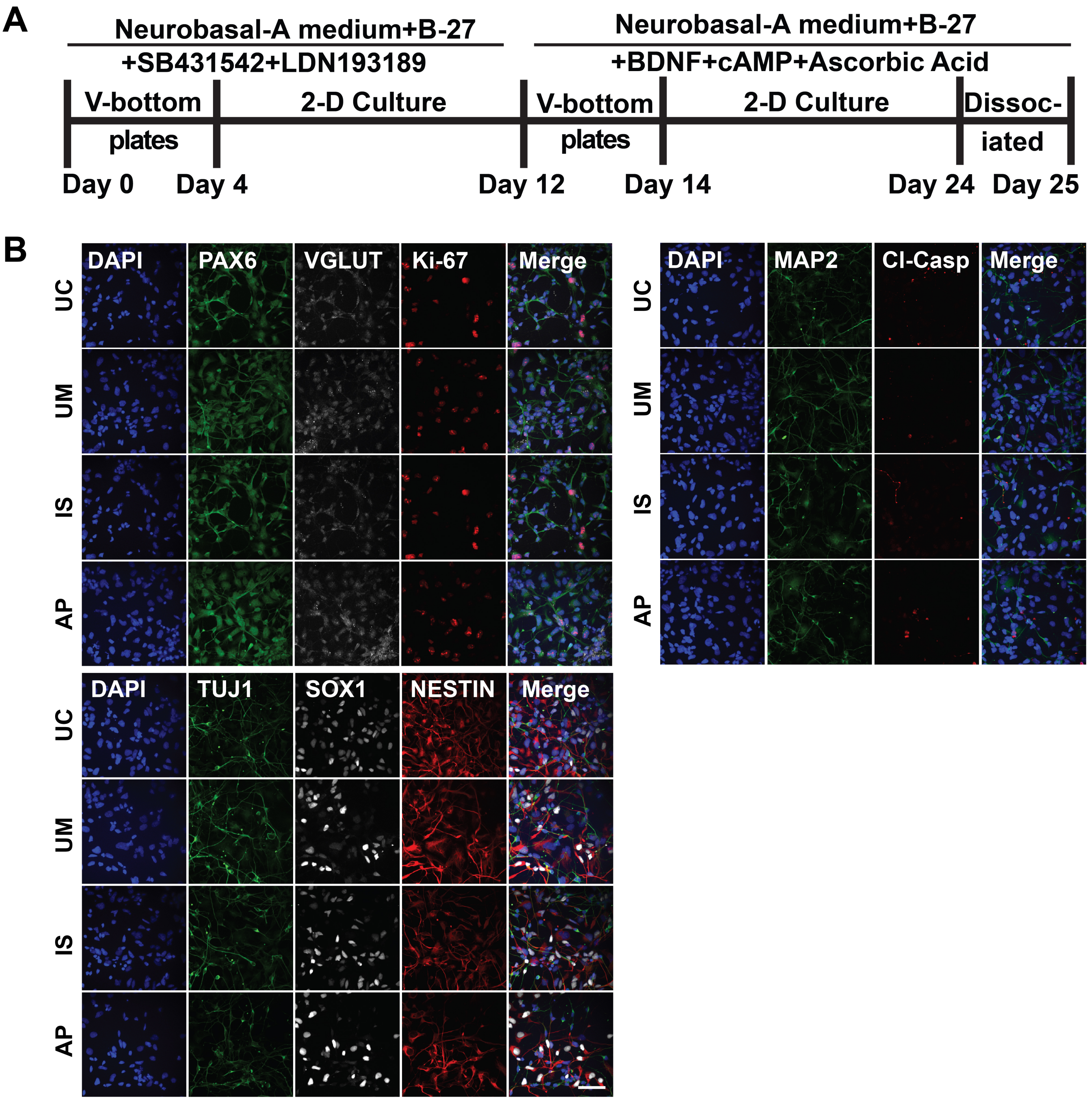
A
Neurobasal-A medium+B-27
+SB431542+LDN193189
Neurobasal-A medium+B-27
+BDNF+cAMP+Ascorbic Acid
V-bottom plates
2-D Culture
V-bottom plates
2-D Culture
Dissoc-iated
Day 0
Day 4
Day 12
Day 14
Day 24
Day 25
B
DAPI
PAX6
VGLUT
Ki-67
Merge
UC
UM
IS
AP
DAPI
MAP2
Cl-Casp
Merge
DAPI
TUJ1
SOX1
NESTIN
Merge

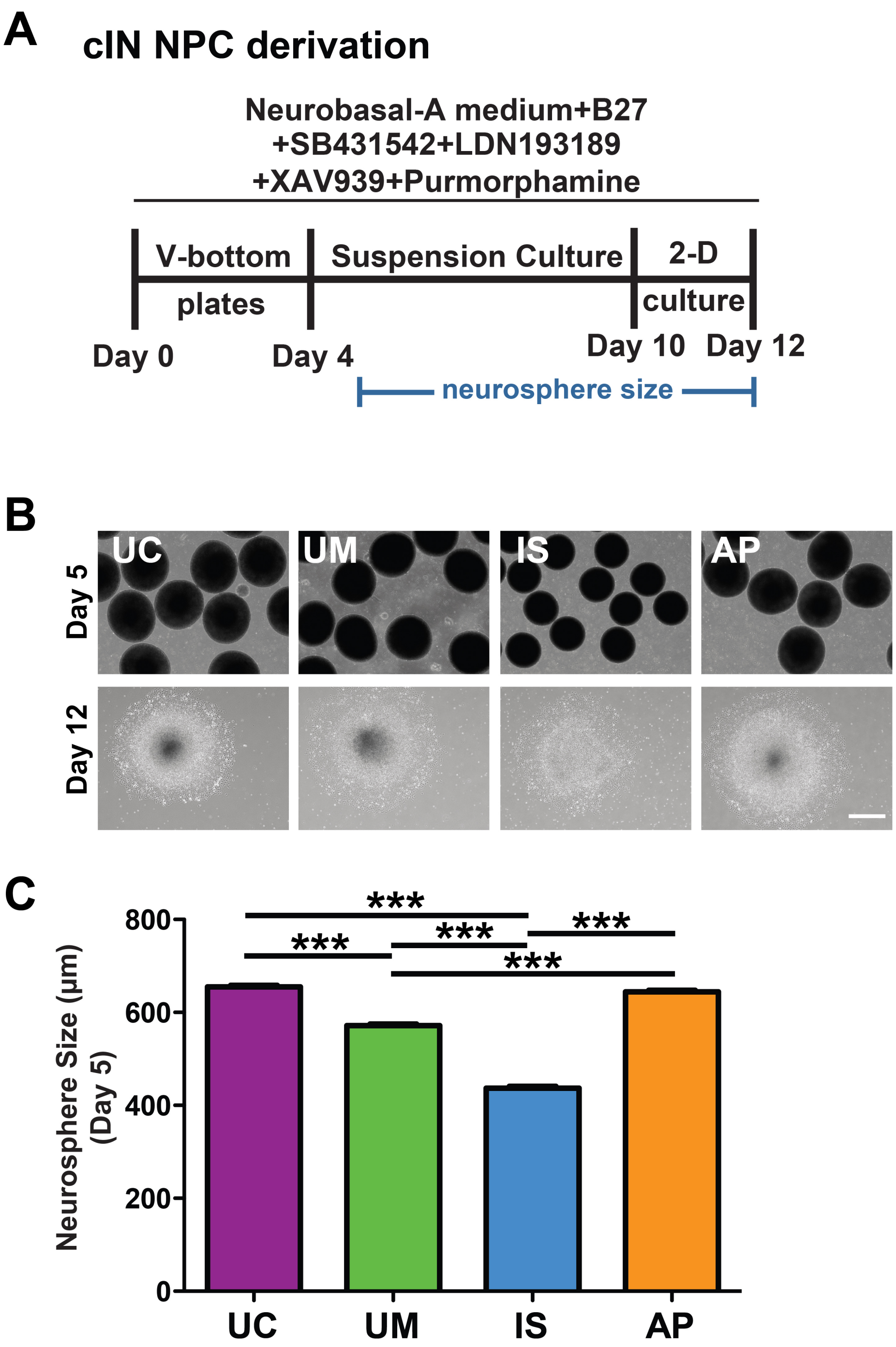
A
cIN NPC derivation
Neurobasal-A medium+B27
+SB431542+LDN193189
+XAV939+Purmorphamine
V-bottom
plates
Suspension Culture
2-D
culture
Day 0
Day 4
Day 10
Day 12
neurosphere size
B
UC
UM
IS
AP
Day 5
Day 12
C
Neurosphere Size (μm)
(Day 5)
800
600
400
200
0
***
UC
UM
IS
AP

S. Fig. S5

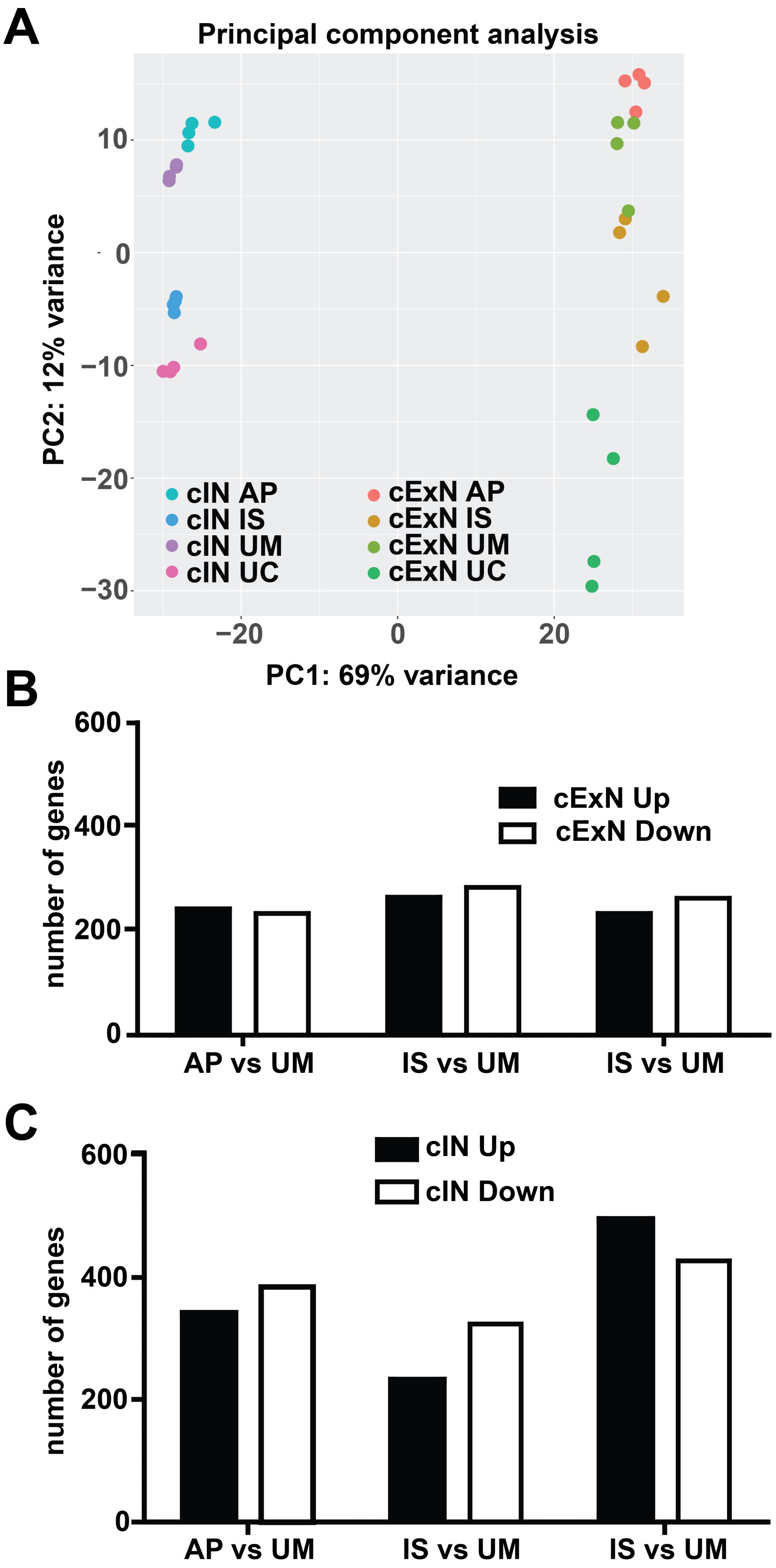
A
Principal component analysis
PC2: 12% variance
PC1: 69% variance
10
0
−10
−20
−30
−20
0
20
cIN AP
cIN IS
cIN UM
cIN UC
cExN AP
cExN IS
cExN UM
cExN UC
B
number of genes
600
400
200
0
cExN Up
cExN Down
AP vs UM
IS vs UM
IS vs UM
C
number of genes
600
400
200
0
cIN Up
cIN Down
AP vs UM
IS vs UM
IS vs UM

S. Fig. S6

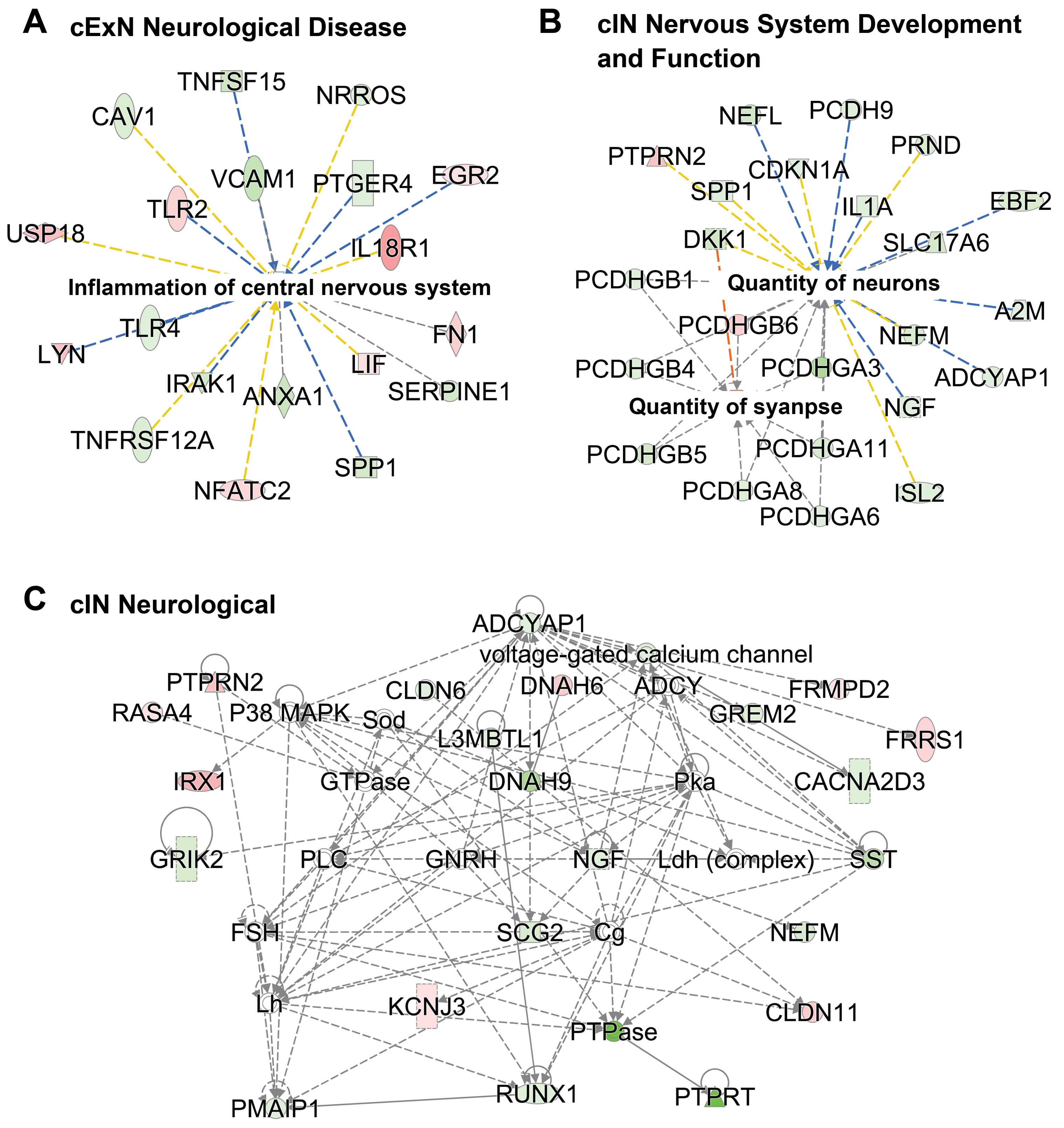
A
cExN Neurological Disease
TNFSF15
NRROS
CAV1
VCAM1
PTGER4
EGR2
TLR2
USP18
IL18R1
Inflammation of central nervous system
TLR4
LYN
FN1
LIF
IRAK1
ANXA1
SERPINE1
TNFRSF12A
SPP1
NFATC2
B
cIN Nervous System Development and Function
NEFL
PCDH9
PRND
PTPRN2
CDKN1A
SPP1
IL1A
EBF2
DKK1
SLC17A6
PCDHGB1
Quantity of neurons
A2M
PCDHGB6
NEFM
PCDHGB4
PCDHGA3
ADCYAP1
Quantity of syanpse
NGF
PCDHGB5
PCDHGA11
PCDHGA8
ISL2
PCDHGA6
C
cIN Neurological
ADCYAP1
voltage-gated calcium channel
PTPRN2
CLDN6
DNAH6
ADCY
FRMPD2
RASA4
P38 MAPK
Sod
GREM2
L3MBTL1
FRRS1
IRX1
GTPase
DNAH9
Pka
CACNA2D3
GRIK2
PLC
GNRH
NGF
Ldh (complex)
SST
FSH
SCG2
Cg
NEFM
Lh
KCNJ3
CLDN11
PTPase
PMAIP1
RUNX1
PTPRT

S. Fig. S7

A cExN WGCNA hierarchical clustering

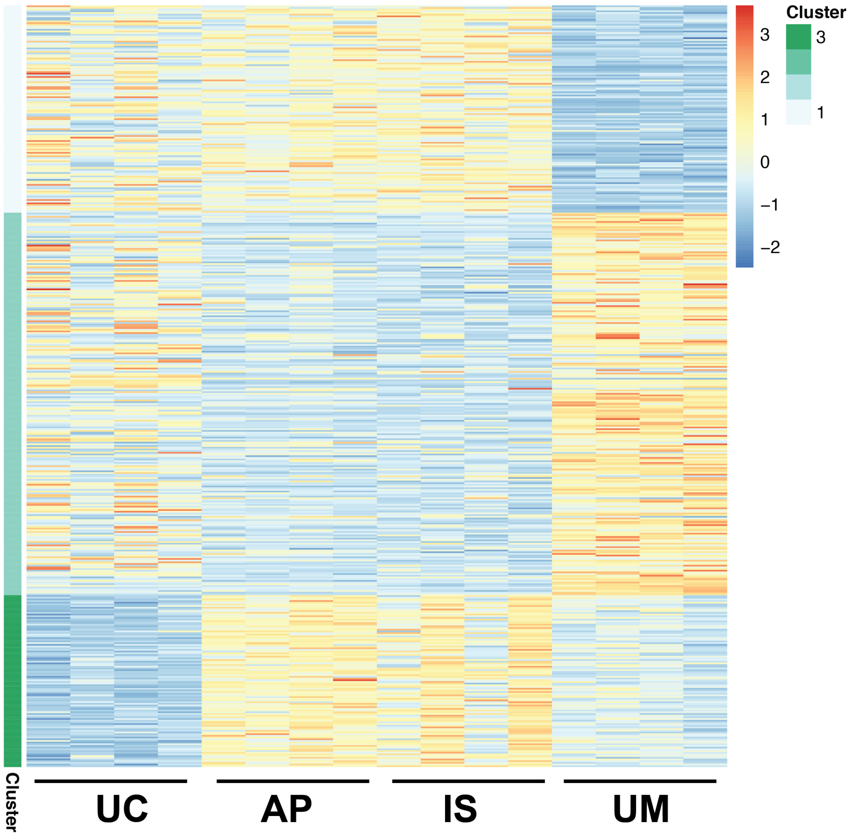

B cIN WGCNA hierarchical clustering

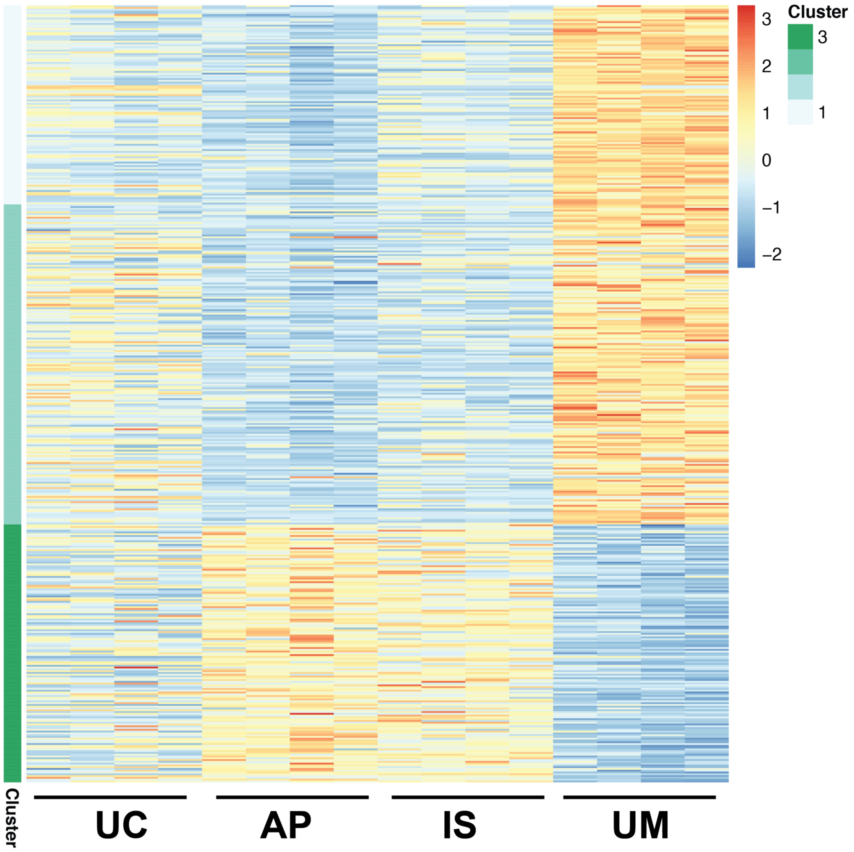

S. Fig. S8

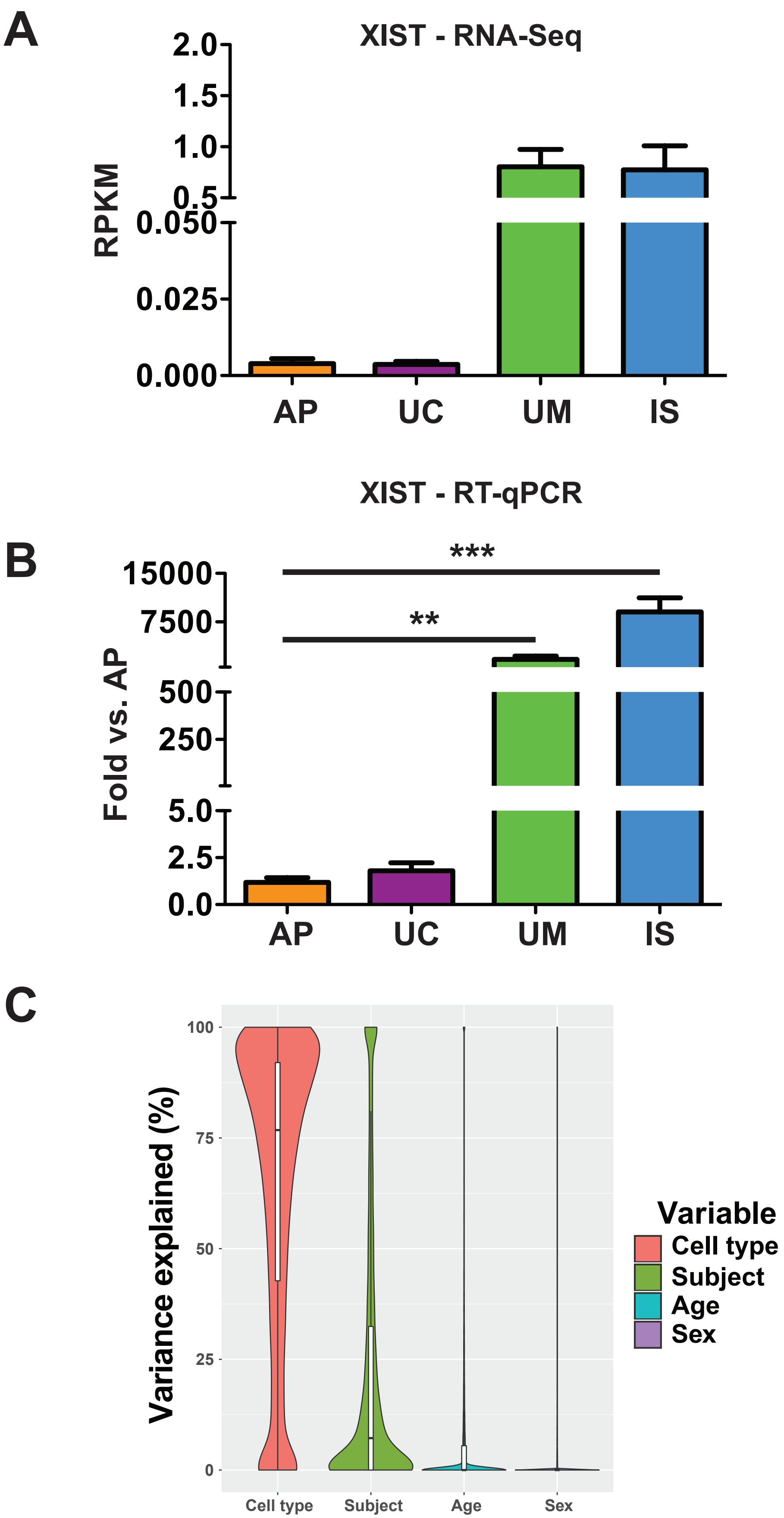

A
XIST - RNA-Seq
RPKM
2.0
1.5
1.0
0.5
0.050
0.025
0.000
AP
UC
UM
IS
XIST - RT-qPCR
B
***
**
15000
7500
500
250
5.0
2.5
0.0
Fold vs. AP
AP
UC
UM
IS
C
Variance explained (%)
100
75
50
25
0
Cell type
Subject
Age
Sex
Variable
Cell type
Subject
Age
Sex
