# Supplementary material for: Cellular and molecular characterization of multiplex autism in human induced pluripotent stem cell-derived neurons
Source: Mol Autism. 2019 Dec 30;10:51. doi: 10.1186/s13229-019-0306-0 (PMC6936127; doi:10.1186/s13229-019-0306-0)
Supplement: Supplementary file 5 — Additional file 5: Table S4. Information about selected DEGs within IPA networks. [file 13229_2019_306_MOESM5_ESM.pdf]

Additional file 5 – Table S4 – Details about selected DEGs within IPA networks.					
**cExN or cIN, IS/AP shared or AP-specific**	**Gene Name**	**Class**	**Network identified**	**Physiological role and function**	**References**
cExN – IS/AP shared	*CHL1*	Adhesion molecule	Behavior (Locomotion), Behavior and Developmental Disorder	Nervous system development and synaptic plasticity.	(1, 2)
	KCNC1	Ion channel	Behavior (Locomotion)	Potassium channel, loss associated with epilepsy and ID.	(3)
	SPP1	ECM protein	Behavior (Locomotion), Behavior and Developmental Disorder, Neurological Disease (Inflammation of Central Nervous System)	Neuroprotective, enhances NSC survival and proliferation.	(4, 5)
	TPH1	Enzyme	Behavior (Locomotion), Behavior and Developmental Disorder	Serotonin biosynthesis, mutated in schizophrenia and other neuropsychiatric disorders.	(6, 7)
	GAS7	PCH protein	Behavior and Developmental Disorder	Adaptor protein; regulate cytoskeleton/membrane dynamics. Involved in neurite outgrowth.	(8, 9)
	XYLT1	Xylosyl transferase	Behavior and Developmental Disorder	Involved in proteoglycan synthesis, which has a role in neuronal migration.	(10, 11)
	SPATA18	Mitochondria-eating protein	Behavior and Developmental Disorder	Mitochondrial response to oxidative stress.	(12)
	CHCHD2	Mitochondrial protein	Behavior and Developmental Disorder	Regulates metabolism and scavenging reactive oxygen species. Negative regulator of mitochondria-mediated apoptosis.	(13)
	VCAM1	Adhesion	Neurological Disease (Inflammation of Central Nervous System)	Maintains NSC identity and adult NSC niche.	(14, 15)
	ANXA1	Annexin	Neurological Disease (Inflammation of Central Nervous System)	Proliferation, differentiation, apoptosis. Anti-inflammatory. Recurrent duplications associated with ASD.	(16-18)
	SERPINE1	Serine proteinase inhibitor	Neurological Disease (Inflammation of Central Nervous System)	Part of MET signaling cascade, which has been associated with ASD. Role in brain not known. Upregulated in human	(19, 20)

				NSCs versus other brain tissue.	
	TLR4 and TLR2	Toll-like receptor	Neurological Disease (Inflammation of Central Nervous System) and Behavior (Locomotion)	Neuronal differentiation and survival.	(21, 22)
	IRAK1	Kinase – member of Toll/IL-1-receptor family	Neurological Disease (Inflammation of Central Nervous System)	Might contribute to neuroprotection.	(23)
cExN – AP-specific	*ERBB4*	EGF receptor tyrosine kinase	Behavior (Memory and Learning) and Nervous System Development and Function (Differentiation of Neurons)	Proliferation, differentiation, migration, and survival of neural cells.	(24, 25)
	FOXB1	Transcription factor	Behavior (Memory and Learning)	Expressed in neural tube, involved in anterior-posterior patterning and in neural development during embryogenesis	(26, 27)
	COMT	Catechol-O-methyltransferase	Behavior (Memory and Learning)	Breaks down dopamine to maintain normal physiological levels in the prefrontal cortex.	(28)
	SLC8A2	Sodium/ calcium exchanger	Behavior (Memory and Learning)	Involved in synaptic plasticity.	(29)
	EMX1	Transcription factor	Nervous System Development and Function (Differentiation of Neurons)	Central role in neural development.	(30)
cIN – IS/AP shared	*KCNJ3* and *KCNJ2*	Ion channels	Behavior (Behavior), Neurological	Behavior, mood disorder, and motor coordination.	(31-34)
	CACNA2D3	Ion channel	Behavior (Behavior), Neurological, Psychological Disorder (Anxiety Disorders)	Mood and cognition.	(35)
	SCN9A	Ion channel	Psychological Disorder (Anxiety Disorders and Depressive Disorder)	Excitability of sensory and cortical neurons.	(36)
	ADCYAP1	Neuropeptide	Behavior (Learning, Cognition, and Behavior), Nervous System Development and Function (Quantity of Neurons)	Regulation of psychomotor and sensory motor behavior and social interactions.	(37, 38)

	GRIK2, GRIK3	Receptors	Psychological Disorder (Mood Disorders), Behavior (Behavior), Neurological	Motor activity and habituation.	(31, 39, 40)
	SST	Calcium binding protein	Behavior (Learning, Cognition, and Behavior), Neurological	Mood disturbances.	(41)
	PCDH9 and *PCDHGA11*	Adhesion molecules	Behavior (Learning, Cognition, and Behavior), Nervous System Development and Function (Quantity of Synapse and Quantity of Neurons)	Learning, memory, behavior, neuronal migration, axonal growth, and synaptic function.	(42-46)
	SYT4	Transcription factor	Behavior (Learning and Cognition)	Synaptic transmission and mental retardation.	(47, 48)
cIN - AP-specific	*GRIA1 and GRIA2*	Receptors	Behavior (Behavior) and Nervous System Development and Function (Synaptic Transmission)	Synaptic structural and functional plasticity.	(49)
	GAP43	Gap junction	Nervous System Development and Function (Development of Neurons)	Stress and abnormal behavior.	(50)
	ARC	Cytoskeleton protein	Nervous System Development and Function (Synaptic Transmission), Behavior (Behavior and Cognition)	Synaptic plasticity and memory.	(51)
	MYT1L	Transcription factor	Nervous System Development and Function (Development of Neurons)	Brain development and intellectual disability.	(52, 53)
	CNTN1 and CNTN2	Adhesion molecules	Behavior (Behavior and Cognition), Nervous System Development (Development of Neurons)	Nervous system development.	(54)

References:

1. Huang X, Sun J, Rong W, Zhao T, Li DH, Ding X, et al. Loss of cell adhesion molecule CHL1 improves homeostatic adaptation and survival in hypoxic stress. Cell Death Dis. 2013;4:e768.
2. Li C, Liu C, Zhou B, Hu C, Xu X. Novel microduplication of CHL1 gene in a patient with autism spectrum disorder: a case report and a brief literature review. Mol Cytogenet. 2016;9:51.
3. Poirier K, Viot G, Lombardi L, Jauny C, Billuart P, Bienvenu T. Loss of Function of KCNC1 is associated with intellectual disability without seizures. Eur J Hum Genet. 2017;25(5):560-4.
4. Wagner PJ, Park HR, Wang Z, Kirchner R, Wei Y, Su L, et al. In Vitro Effects of Lead on Gene Expression in Neural Stem Cells and Associations between Up-regulated Genes and Cognitive Scores in Children. Environ Health Perspect. 2017;125(4):721-9.
5. Rabenstein M, Hucklenbroich J, Willuweit A, Ladwig A, Fink GR, Schroeter M, et al. Osteopontin mediates survival, proliferation and migration of neural stem cells through the chemokine receptor CXCR4. Stem Cell Res Ther. 2015;6:99.
6. Saetre P, Lundmark P, Wang A, Hansen T, Rasmussen HB, Djurovic S, et al. The tryptophan hydroxylase 1 (TPH1) gene, schizophrenia susceptibility, and suicidal behavior: a multi-centre case-control study and meta-analysis. Am J Med Genet B Neuropsychiatr Genet. 2010;153B(2):387-96.
7. Nakamura K, Hasegawa H. Developmental role of tryptophan hydroxylase in the nervous system. Mol Neurobiol. 2007;35(1):45-54.
8. You JJ, Lin-Chao S. Gas7 functions with N-WASP to regulate the neurite outgrowth of hippocampal neurons. J Biol Chem. 2010;285(15):11652-66.
9. Zhang Z, Zheng F, You Y, Ma Y, Lu T, Yue W, et al. Growth arrest specific gene 7 is associated with schizophrenia and regulates neuronal migration and morphogenesis. Mol Brain. 2016;9(1):54.
10. Maeda N. Proteoglycans and neuronal migration in the cerebral cortex during development and disease. Front Neurosci. 2015;9:98.
11. Schreml J, Durmaz B, Cogulu O, Keupp K, Beleggia F, Pohl E, et al. The missing "link": an autosomal recessive short stature syndrome caused by a hypofunctional XYLT1 mutation. Hum Genet. 2014;133(1):29-39.
12. Wang DB, Kinoshita C, Kinoshita Y, Morrison RS. p53 and mitochondrial function in neurons. Biochim Biophys Acta. 2014;1842(8):1186-97.
13. Liu Y, Clegg HV, Leslie PL, Di J, Tollini LA, He Y, et al. CHCHD2 inhibits apoptosis by interacting with Bcl-x L to regulate Bax activation. Cell Death Differ. 2015;22(6):1035-46.
14. Hu XL, Chen G, Zhang S, Zheng J, Wu J, Bai QR, et al. Persistent Expression of VCAM1 in Radial Glial Cells Is Required for the Embryonic Origin of Postnatal Neural Stem Cells. Neuron. 2017;95(2):309-25 e6.
15. Kokovay E, Wang Y, Kusek G, Wurster R, Lederman P, Lowry N, et al. VCAM1 is essential to maintain the structure of the SVZ niche and acts as an environmental sensor to regulate SVZ lineage progression. Cell Stem Cell. 2012;11(2):220-30.
16. Solito E, McArthur S, Christian H, Gavins F, Buckingham JC, Gillies GE. Annexin A1 in the brain--undiscovered roles? Trends Pharmacol Sci. 2008;29(3):135-42.
17. Correia CT, Conceicao IC, Oliveira B, Coelho J, Sousa I, Sequeira AF, et al. Recurrent duplications of the annexin A1 gene (ANXA1) in autism spectrum disorders. Mol Autism. 2014;5(1):28.
18. Parente L, Solito E. Annexin 1: more than an anti-phospholipase protein. Inflamm Res. 2004;53(4):125-32.

19. Campbell DB, Li C, Sutcliffe JS, Persico AM, Levitt P. Genetic evidence implicating multiple genes in the MET receptor tyrosine kinase pathway in autism spectrum disorder. Autism Res. 2008;1(3):159-68.
20. Sandberg CJ, Vik-Mo EO, Behnan J, Helseth E, Langmoen IA. Transcriptional profiling of adult neural stem-like cells from the human brain. PLoS One. 2014;9(12):e114739.
21. Grasselli C, Ferrari D, Zalfa C, Soncini M, Mazzoccoli G, Facchini FA, et al. Toll-like receptor 4 modulation influences human neural stem cell proliferation and differentiation. Cell Death Dis. 2018;9(3):280.
22. Ma Y, Haynes RL, Sidman RL, Vartanian T. TLR8: an innate immune receptor in brain, neurons and axons. Cell Cycle. 2007;6(23):2859-68.
23. Rao N, Nguyen S, Ngo K, Fung-Leung WP. A novel splice variant of interleukin-1 receptor (IL-1R)-associated kinase 1 plays a negative regulatory role in Toll/IL-1R-induced inflammatory signaling. Mol Cell Biol. 2005;25(15):6521-32.
24. Perez-Garcia CG. ErbB4 in Laminated Brain Structures: A Neurodevelopmental Approach to Schizophrenia. Front Cell Neurosci. 2015;9:472.
25. Yau HJ, Wang HF, Lai C, Liu FC. Neural development of the neuregulin receptor ErbB4 in the cerebral cortex and the hippocampus: preferential expression by interneurons tangentially migrating from the ganglionic eminences. Cereb Cortex. 2003;13(3):252-64.
26. Zhang Y, Hoxha E, Zhao T, Zhou X, Alvarez-Bolado G. Foxb1 Regulates Negatively the Proliferation of Oligodendrocyte Progenitors. Front Neuroanat. 2017;11:53.
27. Takebayashi-Suzuki K, Kitayama A, Terasaka-Iioka C, Ueno N, Suzuki A. The forkhead transcription factor FoxB1 regulates the dorsal-ventral and anterior-posterior patterning of the ectoderm during early Xenopus embryogenesis. Dev Biol. 2011;360(1):11-29.
28. Chen J, Lipska BK, Halim N, Ma QD, Matsumoto M, Melhem S, et al. Functional analysis of genetic variation in catechol-O-methyltransferase (COMT): effects on mRNA, protein, and enzyme activity in postmortem human brain. Am J Hum Genet. 2004;75(5):807-21.
29. Jeon D, Yang YM, Jeong MJ, Philipson KD, Rhim H, Shin HS. Enhanced learning and memory in mice lacking Na+/Ca2+ exchanger 2. Neuron. 2003;38(6):965-76.
30. Kobeissy FH, Hansen K, Neumann M, Fu S, Jin K, Liu J. Deciphering the Role of Emx1 in Neurogenesis: A Neuroproteomics Approach. Front Mol Neurosci. 2016;9:98.
31. Pravetoni M, Wickman K. Behavioral characterization of mice lacking GIRK/Kir3 channel subunits. Genes Brain Behav. 2008;7(5):523-31.
32. Mayfield J, Blednov YA, Harris RA. Behavioral and Genetic Evidence for GIRK Channels in the CNS: Role in Physiology, Pathophysiology, and Drug Addiction. Int Rev Neurobiol. 2015;123:279-313.
33. Guglielmi L, Servettini I, Caramia M, Catacuzzeno L, Franciolini F, D'Adamo MC, et al. Update on the implication of potassium channels in autism: K(+) channelautism spectrum disorder. Front Cell Neurosci. 2015;9:34.
34. Binda A, Rivolta I, Villa C, Chisci E, Beghi M, Cornaggia CM, et al. A Novel KCNJ2 Mutation Identified in an Autistic Proband Affects the Single Channel Properties of Kir2.1. Front Cell Neurosci. 2018;12:76.
35. Kabir ZD, Lee AS, Rajadhyaksha AM. L-type Ca(2+) channels in mood, cognition and addiction: integrating human and rodent studies with a focus on behavioural endophenotypes. J Physiol. 2016;594(20):5823-37.

36. Rubinstein M, Patowary A, Stanaway IB, McCord E, Nesbitt RR, Archer M, et al. Association of rare missense variants in the second intracellular loop of NaV1.7 sodium channels with familial autism. Mol Psychiatry. 2018;23(2):231-9.
37. Nicot A, Otto T, Brabet P, Dicicco-Bloom EM. Altered social behavior in pituitary adenylate cyclase-activating polypeptide type I receptor-deficient mice. J Neurosci. 2004;24(40):8786-95.
38. Tanaka K, Shintani N, Hashimoto H, Kawagishi N, Ago Y, Matsuda T, et al. Psychostimulant-induced attenuation of hyperactivity and prepulse inhibition deficits in Adcyap1-deficient mice. J Neurosci. 2006;26(19):5091-7.
39. Contractor A, Mulle C, Swanson GT. Kainate receptors coming of age: milestones of two decades of research. Trends Neurosci. 2011;34(3):154-63.
40. Kim SA, Kim JH, Park M, Cho IH, Yoo HJ. Family-based association study between GRIK2 polymorphisms and autism spectrum disorders in the Korean trios. Neurosci Res. 2007;58(3):332-5.
41. Lin LC, Sibille E. Reduced brain somatostatin in mood disorders: a common pathophysiological substrate and drug target? Front Pharmacol. 2013;4:110.
42. Chen J, Yu S, Fu Y, Li X. Synaptic proteins and receptors defects in autism spectrum disorders. Front Cell Neurosci. 2014;8:276.
43. Tsai NP, Huber KM. Protocadherins and the Social Brain. Biol Psychiatry. 2017;81(3):173-4.
44. Washbourne P, Dityatev A, Scheiffele P, Biederer T, Weiner JA, Christopherson KS, et al. Cell adhesion molecules in synapse formation. J Neurosci. 2004;24(42):9244-9.
45. Wang X, Weiner JA, Levi S, Craig AM, Bradley A, Sanes JR. Gamma protocadherins are required for survival of spinal interneurons. Neuron. 2002;36(5):843-54.
46. Bruining H, Matsui A, Oguro-Ando A, Kahn RS, Van't Spijker HM, Akkermans G, et al. Genetic Mapping in Mice Reveals the Involvement of Pcdh9 in Long-Term Social and Object Recognition and Sensorimotor Development. Biol Psychiatry. 2015;78(7):485-95.
47. Ting JT, Kelley BG, Sullivan JM. Synaptotagmin IV does not alter excitatory fast synaptic transmission or fusion pore kinetics in mammalian CNS neurons. J Neurosci. 2006;26(2):372-80.
48. Buysse K, Menten B, Oostra A, Tavernier S, Mortier GR, Speleman F. Delineation of a critical region on chromosome 18 for the del(18)(q12.2q21.1) syndrome. Am J Med Genet A. 2008;146A(10):1330-4.
49. Ramanathan S, Woodroffe A, Flodman PL, Mays LZ, Hanouni M, Modahl CB, et al. A case of autism with an interstitial deletion on 4q leading to hemizygosity for genes encoding for glutamine and glycine neurotransmitter receptor sub-units (AMPA 2, GLRA3, GLRB) and neuropeptide receptors NPY1R, NPY5R. BMC Med Genet. 2004;5:10.
50. Zaccaria KJ, Lagace DC, Eisch AJ, McCasland JS. Resistance to change and vulnerability to stress: autistic-like features of GAP43-deficient mice. Genes Brain Behav. 2010;9(8):985-96.
51. Korb E, Finkbeiner S. Arc in synaptic plasticity: from gene to behavior. Trends Neurosci. 2011;34(11):591-8.
52. Blanchet P, Bebin M, Bruet S, Cooper GM, Thompson ML, Duban-Bedu B, et al. MYT1L mutations cause intellectual disability and variable obesity by dysregulating gene expression and development of the neuroendocrine hypothalamus. PLoS Genet. 2017;13(8):e1006957.
53. Stevens SJ, van Ravenswaaij-Arts CM, Janssen JW, Klein Wassink-Ruiter JS, van Essen AJ, Dijkhuizen T, et al. MYT1L is a candidate gene for intellectual disability in patients with 2p25.3 (2pter) deletions. Am J Med Genet A. 2011;155A(11):2739-45.

54. Mohebiany AN, Harroch S, Bouyain S. New insights into the roles of the contactin cell adhesion molecules in neural development. Adv Neurobiol. 2014;8:165-94.
